# Generative AI Use and Depressive Symptoms Among US Adults

**DOI:** 10.1001/jamanetworkopen.2025.54820

**Published:** 2026-01-21

**Authors:** Roy H. Perlis, Faith M. Gunning, Ata A. Uslu, Mauricio Santillana, Matthew A. Baum, James N. Druckman, Katherine Ognyanova, David Lazer

**Affiliations:** 1 Center for Quantitative Health, Massachusetts General Hospital, Boston; 2 Department of Psychiatry, Weill-Cornell Medical School, New York, New York; 3 Network Science Institute, Northeastern University, Boston, Massachusetts; 4 Institute for Quantitative Social Science, Harvard University, Boston, Massachusetts; 5 John F. Kennedy School of Government, Harvard University, Cambridge, Massachusetts; 6 Department of Political Science, University of Rochester, Rochester, New York; 7 Department of Communication, School of Communication and Information, Rutgers University, New Brunswick, New Jersey; 8 Department of Psychiatry, Massachusetts General Hospital, Boston; 9 Department of Government, Harvard University, Cambridge, Massachusetts

## Abstract

**Importance:**

Generative artificial intelligence (AI) has rapidly entered mainstream use in the US, but its association with mental health has not been characterized.

**Objective:**

To examine the associations of the extent and type of generative AI use among US adults with negative affective symptoms in a large, nationally representative sample.

**Design, Setting, and Participants:**

This survey study used data from a 50-state US internet nonprobability survey conducted between April and May 2025. Survey respondents were aged 18 years and older. Data were analyzed in August 2025.

**Exposure:**

Participants self-reported generative AI and social media use.

**Main Outcomes and Measures:**

The outcome of interest, negative affect, was measured using the Patient Health Questionnaire 9-item (PHQ-9).

**Results:**

There were 20 847 unique participants, with mean (SD) age 47.3 (17.1) years and 10 327 (49.5%) female, 10 386 (49.8%) male, and 134 (0.6%) nonbinary participants; 2152 participants (10.3%) reported using AI at least daily, including 1053 participants (5.1%) who reported daily use and 1099 participants (5.3%) who reported use multiple times per day. Among participants who used daily or more frequently, 1033 (48.0%) reported use for work, 246 (11.4%) for school, and 1875 (87.1%) for personal applications. In survey-weighted regression models, daily or more frequent AI use was significantly more common among men, younger adults, those with higher education and income, and those in urban settings. Greater AI use was associated with greater levels of depressive symptoms in sociodemographic-adjusted regression models: (daily use: β = 1.08 [95% CI, 0.55-1.62]; multiple times per day: β = 0.86 [95% CI, 0.35-1.37]) compared with nonuse, and with greater likelihood of reporting at least moderate depressive symptoms (odds ratio [OR], 1.29 [95% CI, 1.15-1.46]); similar patterns were observed for anxiety and irritability. The highest estimates were observed among individuals using AI for personal use (β = 0.31 [95% CI, 0.10-0.52]) and those aged 25 to 44 years (β = 1.22 [95% CI, 0.70-1.74]) or 45 to 64 years (β = 1.38 [95% CI, 0.72-2.05]).

**Conclusions and Relevance:**

This survey study found that AI use was significantly associated with greater depressive symptoms, with magnitude of differences varying by age group. Further work is needed to understand whether these associations are causal and explain heterogeneous effects.

## Key Points

**Question:**

Are greater levels of generative artificial intelligence (AI) use by US adults associated with greater levels of depressive symptoms?

**Findings:**

In this survey study of 20 847 US adults, 10.3% reported daily use of generative AI, including 5.3% who used it multiple times per day. Greater levels of AI use were associated with modest increases in depressive symptoms, with odds of at least moderate depression 30% greater among those with daily use, particularly among younger users.

**Meaning:**

These findings suggest there is a need to understand the nature and mechanism of this association and its heterogeneity across age-defined subgroups.

## Teaser

This survey study examines the associations of generative artificial intelligence (AI) use with negative affective symptoms in a large nationally representative sample of US adults.

## Introduction

Use of generative artificial intelligence (AI) has increased massively since the introduction of ChatGPT 3.5 (OpenAI) in late 2022.^1^ Along with enthusiasm for the potential benefits of these tools,^2^ anecdotal evidence of potential harms to mental health has emerged, with suggestions that use of chatbots may precipitate or exacerbate delusions in vulnerable individuals,^3^ and even contribute to risk for suicide attempts in rare cases.^4^ An report from Phang et al^5^ of more than 3 million generative AI conversations suggested that a subset of users with prolonged daily use demonstrated evidence of dependence and reduced social engagement, while briefer interactions were associated with improved well-being. A similar pattern was observed in a survey of college students, with greater chatbot use associated with greater depressive symptoms.^6^ Conversely, a randomized trial suggested benefit for a chatbot applying a language model specifically trained to discuss mental health symptoms,^7^ suggesting that the nature and context of use may be important to consider.

The pattern of concerns with AI has paralleled those arising from social media use. The latter has been associated with negative affective states, including depression, anxiety, and irritability.^8^ A meta-analysis of frequent use among adolescents and young adults found moderate associations of social media use with depression and stress^9^; an earlier systematic review of studies examining social network sites found both potentially harmful and beneficial outcomes.^10^ On the other hand, whether such associations are causal has not been established: a small number of longitudinal studies suggest that social media use may precede such states^11,12^, while a meta-analysis did not find a benefit associated with social media abstinence.^13^ Two randomized clinical trials suggested that brief discontinuation of social media may diminish depressive and anxious symptoms,^14^ although these effects were extremely modest in magnitude.^15^ Taken together, this literature suggests potential harms in some contexts, but effects that may be heterogeneous.

In light of these parallels between social media and generative AI, the initial signals of potential risk associated with AI merits more systematic investigation. In this study, we sought to understand the association between AI use and mood symptoms in a very large and nationally representative group of US adults, drawing on data from a large, 50-state survey that assessed a broad range of behaviors and attitudes. We examined individuals’ use of AI and its associations with measures of mental health, and the extent to which such use correlated with other social media use. We further considered whether these associations varied across sociodemographic groups, as a first step toward future studies aimed at identifying more vulnerable or resilient populations.

## Methods

### Study Design

We analyzed data drawn from 1 wave of the Civic Health and Institutions Project (CHIP-50) study,^16^ previously the COVID States Project, a nonprobability^17^ internet survey conducted through a commercial survey panel aggregator (PureSpectrum). The wave (wave 35) that included questions regarding AI use was conducted between April 20 and May 27, 2025. Survey participants were aged 18 years and older residing in 1 of the 50 US states or the District of Columbia who opted in to a general opinion survey. The survey used quotas for each state to balance for age, gender, and racial and ethnic group participation. Written online consent was provided by all participants prior to answering survey questions. The survey protocol was determined to be exempt by the Harvard University institutional review board per Common Rule Exemption Category 2. The survey was conducted and results are presented in accordance with American Association for Public Opinion Research (AAPOR) reporting guideline.

### Measures

#### AI and Social Media Use

To assess frequency of AI use, all participants were asked, “How often do you use the following technologies or products? - Artificial intelligence (AI)”; options included never, once or twice, about once a month, about once a week, multiple times a week, every day, and multiple times a day. We treated this variable ordinally, and for descriptive purposes then dichotomized to at least daily or not at least daily. To account for the possibility that some respondents would recognize other terms (eg, generative AI, large language models), we asked the same question with that phrasing, yielding nearly identical results. Participants were also asked, “For what purposes do you use the following technologies or products? (Please select all that apply) - Artificial intelligence” with the option to select personal use, for work, and for school.

Participants were similarly asked how often they use individual social media platforms, with options ranging from never to many times every day. For purposes of analysis, we examined maximum use across all platforms. As with AI use, frequency was dichotomized to at least daily or less than daily for secondary analyses. An additional question asked about posting frequency, by platform, with the same anchor points.

#### Assessment of Negative Affective Symptoms

We quantified depressive symptom severity with the 9-item Patient Health Questionnaire (PHQ-9),^18,19^ which includes individual diagnostic criteria for major depressive disorder in the *Diagnostic and Statistical Manual of Mental Disorders* (Fifth Edition) (*DSM-5*); respondents rate each symptom in terms of frequency over the prior 2 weeks on a 0 to 3 Likert-type scale (0 indicates not at all; 3, nearly every day) yielding a total score from 0 to 27. Scoring 10 or greater represents moderate or greater depressive symptoms and is typically considered a threshold for treatment referral.^18,19^ On this scale, item 9 assesses thoughts of death or suicide, inquiring about frequency of “[t]houghts that you would be better off dead, or of hurting yourself.”^20^ We quantified anxiety with the 2-item Generalized Anxiety Disorder screen (GAD-2), derived from the 7-item version^21^ of this screening measure; a 3 or greater on this scale represents probable generalized anxiety. We quantified irritability via the Brief Irritability Test (BITe-5), which includes 5 questions beginning with, “Please indicate how often you have felt or behaved in the following ways, during the past two weeks, including today.”^22^ Frequency for each item is reported on a 1 to 6 scale, from never to always, and item scores are summed to yield a total score between 5 and 30. The scale was designed to minimize correlation with depression, anger, and similar constructs,^22^ and it has been used in other survey-based investigations of negative affect.^23^

#### Sociodemographic Variables

We collected sociodemographic features by self-report and categorized them as in our prior survey reports.^8,12,24,25^ These included identifying race and ethnicity from a list, including African American or Black, Asian American, Hispanic, Native American, Pacific Islander, White, or other, with the opportunity to provide a free text self-description. Race and ethnicity were collected to allow us to confirm representativeness of the US population, and are reported in accordance with guidelines for use of such data.^26^ We collapsed Native American, Pacific Islander, and other into a single category to facilitate analysis, as the individual groups were small. We categorized household socioeconomic status in terms of annual household income as less than $25 000, $25 000 to less than $50 000, $50 000 to less than $100 000, or $100 000 or more per year. We determined educational status by asking participants to select from highest level of formal education from a list including some high school or less, high school graduate, some college, undergraduate degree, or graduate degree.

### Statistical Analysis

All analyses were conducted in R software version 4.4.2 (R Project for Statistical Computing). Continuous measures are reported in terms of mean and SD, and categorical measures in terms of proportions and CIs. We applied interlocking poststratification survey weights to approximate national demographic distributions (race and ethnicity, age, gender, educational level, region, and living in urban, suburban, or rural areas) as determined by 2020 US Census American Community Survey results,^27^ as advised for nonprobability samples,^28^ with the R survey package^29^ (version 4.2-1). For all analyses, 2-tailed *P* ≤ .05 was considered to represent statistical significance. Data were analyzed in August 2025.

We began by characterizing frequency of AI use in the cohort as a whole. We used survey-weighted logistic regression to examine association between sociodemographic features and daily AI use for ease of interpretation, complemented by ordinal logistic regression (via polr in R) after confirming proportionality of odds. Models were fit without covariates, then with inclusion of age group, gender, education, annual household income, race and ethnicity, and rural, suburban, or urban setting based on zip code. Next, we examined the association between frequency of AI use and depressive symptoms characterized by PHQ-9 in unadjusted and then sociodemographic-adjusted survey-weighted linear regression models. Secondarily, we examined other negative affective symptoms (anxiety via GAD-2, irritability via BITe-5) with the same approach. To determine whether observed associations could represent a more general association with online activity, we computed polychoric correlations between AI use frequency and social media use or posting frequency, then fit models that included maximum social media use or social media posting as well. We also examined whether type of use modified association with affective symptoms by including indicator variables in the regression model for presence or absence of type of use (personal, work, or school).

Finally, to evaluate potential demographic moderators of AI use association with depressive symptoms, we tested interaction terms for AI use × gender and AI use × age group. Where significant interactions were identified, we fit stratified linear regression models by age group or gender to clarify associations. To frame these results in more clinically interpretable fashion, we then fit survey-weighted logistic regression models to illustrate the differential association with a categorical rather than continuous outcome, namely moderate or severe depression, as defined by PHQ-9.

## Results

In all, there were 20 847 participants, with mean (SD) age 47.3 (17.1) years (SD 17.1); 10 327 (49.5%) identified as female, 10 386 (49.8%) as male, and 134 (0.6%) as nonbinary. Of the full cohort, 3865 participants (18.5%) identified as African American or Black; 980 participants (4.7%) as Asian American, 1360 participants (6.5%) as another race; and 14 642 participants (70.2%) as White. Independent of race, 2977 participants (14.3%) identified as being of Hispanic ethnicity (Table). A total of 2152 participants (10.3%) reported using AI at least daily, including 1053 participants (5.1%) who reported use every day and 1099 participants (5.3%) who reported use multiple times per day. Among daily users, 1033 participants (48.0%) reported use for work, 246 participants (11.4%) for school, and 1875 participants (87.1%) for personal applications.

**Table.** Participant Characteristics by Daily AI Use

Characteristic	Participants by AI use, No. (%)			*P* value
	<1 Time per day (n = 18 695)	≥1 Times per day (n = 2152)	Total (N = 20 847)	
Gender				
Female	9514 (50.9)	813 (37.8)	10 327 (49.5)	<.001
Male	9063 (48.5)	1323 (61.5)	10 386 (49.8)	
Nonbinary	118 (0.6)	16 (0.7)	134 (0.6)	
Age, y				
18-24	1641 (8.8)	214 (9.9)	1855 (8.9)	<.001
25-44	6997 (37.4)	1159 (53.9)	8156 (39.1)	
45-64	6027 (32.2)	624 (29.0)	6651 (31.9)	
≥65	4030 (21.6)	155 (7.2)	4185 (20.1)	
Education				
<High school	705 (3.8)	50 (2.3)	755 (3.6)	<.001
High school graduate	5223 (27.9)	395 (18.4)	5618 (26.9)	
Some college	4691 (25.1)	414 (19.2)	5105 (24.5)	
College degree	6112 (32.7)	833 (38.7)	6945 (33.3)	
Graduate degree	1964 (10.5)	460 (21.4)	2424 (11.6)	
Income, $				
<25 000	4438 (23.7)	338 (15.7)	4776 (22.9)	<.001
25 000 to <50 000	4976 (26.6)	400 (18.6)	5376 (25.8)	
50 000 to <100 000	5860 (31.4)	679 (31.6)	6539 (31.4)	
≥100 000	3418 (18.3)	735 (34.2)	4153 (19.9)	
Race				
African American or Black	3345 (17.9)	520 (24.2)	3865 (18.5)	<.001
Asian American	844 (4.5)	136 (6.3)	980 (4.7)	
Other race^a^	1193 (6.4)	167 (7.8)	1360 (6.5)	
White	13 313 (71.2)	1329 (61.8)	14 642 (70.2)	
Ethnicity				
Hispanic	2541 (13.6)	436 (20.3)	2977 (14.3)	<.001
Non-Hispanic	16 154 (86.4)	1716 (79.7)	17 870 (85.7)	
Urbanicity				
Rural	3348 (17.9)	246 (11.4)	3594 (17.2)	<.001
Suburban	10 148 (54.3)	1027 (47.7)	11 175 (53.6)	
Urban	5199 (27.8)	879 (40.8)	6078 (29.2)	
Social media use				
<Daily	15 014 (80.3)	1686 (78.3)	16 700 (80.1)	.03
≥Daily	3681 (19.7)	466 (21.7)	4147 (19.9)	
Social media posting				
<Daily	17 763 (95.0)	2048 (95.2)	19 811 (95.0)	.76
≥Daily	932 (5.0)	104 (4.8)	1036 (5.0)	
Frequency of AI use				
Never	11 338 (60.6)	NA	11 338 (54.4)	<.001
Once or twice	2891 (15.5)	NA	2891 (13.9)	
About once a month	1227 (6.6)	NA	1227 (5.9)	
About once a week	1407 (7.5)	NA	1407 (6.7)	
Multiple times a week	1832 (9.8)	NA	1832 (8.8)	
Every day	NA	1053 (48.9)	1053 (5.1)	
Multiple times a day	NA	1099 (51.1)	1099 (5.3)	
Type of AI use				
Personal	6094 (32.6)	1875 (87.1)	7969 (38.2)	<.001
Work	1890 (10.1)	1033 (48.0)	2923 (14.0)	<.001
School	461 (2.5)	246 (11.4)	707 (3.4)	<.001
PHQ-9 score				
≥10	4797 (25.9)	688 (32.0)	5485 (26.5)	<.001
Total, mean (SD)	6.3 (6.7)	7.4 (7.2)	6.4 (6.7)	<.001
GAD-2 total, mean (SD)	1.6 (1.8)	1.8 (2.0)	1.6 (1.9)	<.001
BITe-5 total, mean (SD)	12.8 (5.9)	13.7 (6.4)	12.9 (5.9)	<.001

Abbreviations: AI, artificial intelligence; BITe-5, Brief Irritability Test; GAD-2, Generalized Anxiety Disorder 2-item; PHQ-9, Patient Health Questionnaire 9-item.

^a^ Other race includes participants who indicated Native American, Pacific Islander, or other race.

**Figure 1.**
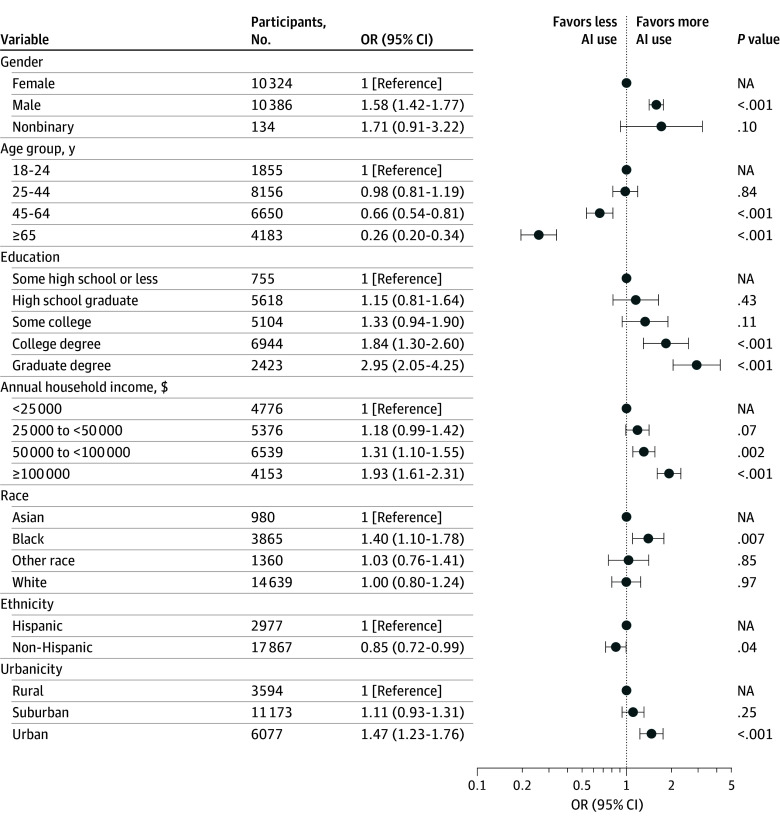
Multivariable Logistic Regression Model of Daily or Greater Artificial Intelligence (AI) Use NA indicates not applicable; OR, odds ratio.

We initially examined sociodemographic features associated with at least daily AI use, individually (Table) and then with survey-weighted multivariable logistic regression models (Figure 1). Overall, AI use was greater among male compared with female respondents (odds ratio [OR], 1.58 [95% CI, 1.42-1.77]), younger individuals (age ≥65 vs age 18-24 years: OR, 0.26 [95% CI, 0.20-0.34]), those with greater levels of education (graduate degree vs some high school or less: OR, 2.95 [95% CI, 2.05-4.25]), greater household income (≥$100 000 vs <$25 000: OR, 1.93 [95% CI, 1.61-2.31]), and those in more urban settings (urban vs rural: OR, 1.47 [95% CI, 1.23-1.76]). To complement these analyses, we repeated them with ordinal logistic regression examining frequency of use, yielding similar results (eFigure 1 in Supplement 1).

**Figure 2.**
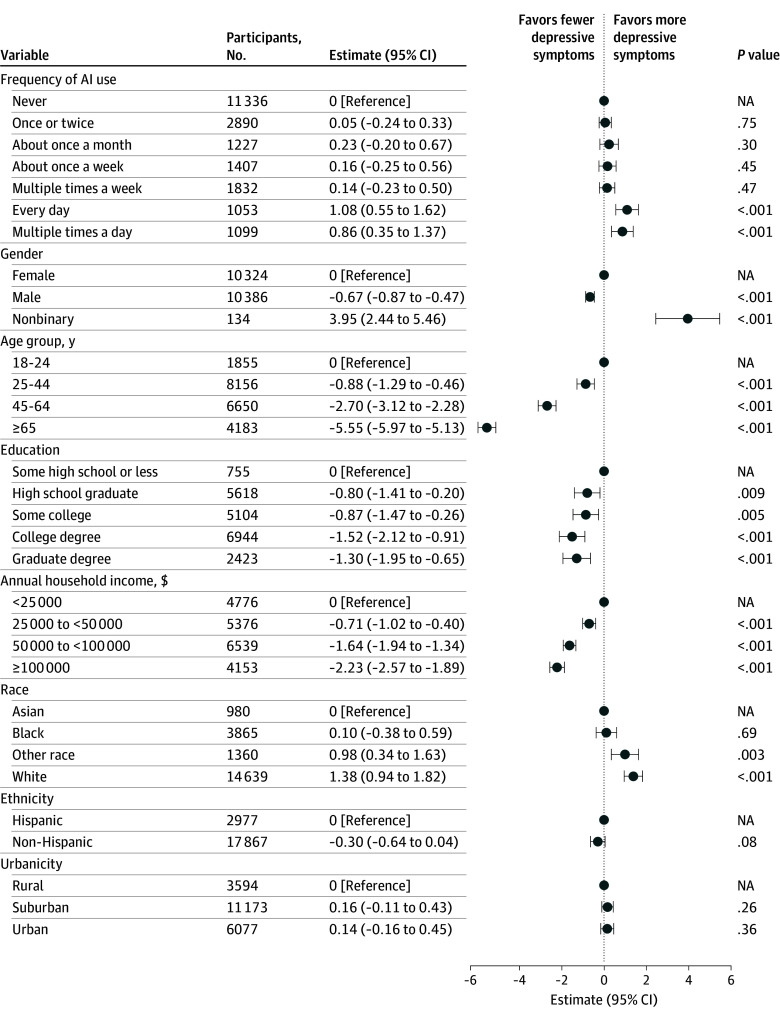
Multivariable Linear Regression Model of Depressive Symptoms AI indicates artificial intelligence; NA, not applicable.

We next examined the association between AI use and negative affective symptoms. In linear regression, extent of use was associated with significantly greater PHQ-9 score, with daily use (β = 1.08 [95% CI, 0.55-1.62]) and multiple times a day use (β = 0.86 [95% CI, 0.35-1.37]) associated with modest but significant increases in depressive symptoms compared with nonuse (Figure 2). Similar associations were observed with anxiety, measured by GAD-2, and irritability, measured by BITe-5 (eFigure 2 and eFigure 3 in Supplement 1).

**Figure 3.**
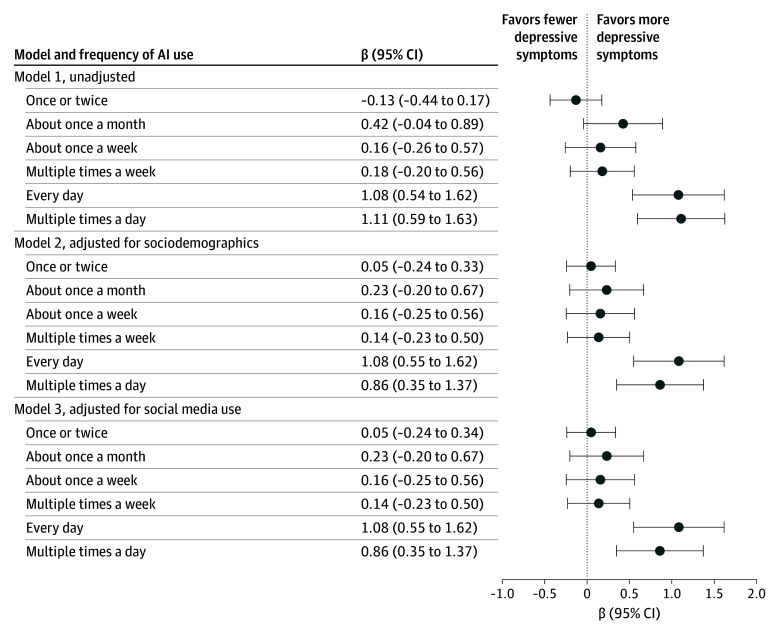
Multivariable Linear Regression Models of Depressive Symptoms by Artificial Intelligence (AI) Use Depressive symptoms were measured using the Patient Health Questionnaire 9-item (PHQ-9). For all comparisons, never AI use was the comparative group. Model 1 was unadjusted; model 2 was adjusted for sociodemographic characteristics; and model 3, sociodemographic characteristics and social media use.

**Figure 4.**
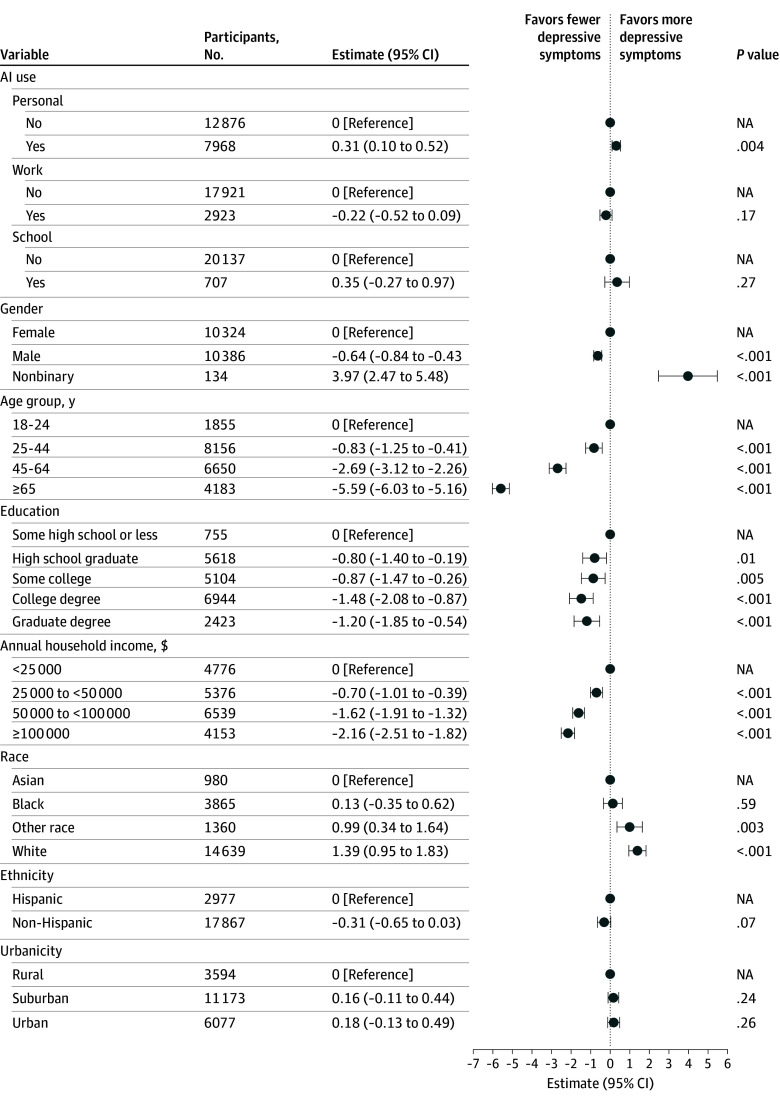
Multivariable Linear Regression Model of Depressive Symptoms Associated With Type of Artificial Intelligence (AI) Use Depressive symptoms were measured using the Patient Health Questionnaire 9-item (PHQ-9). NA indicates not applicable.

We then examined whether the association between AI use and mood might be explained by social media use. Polychoric correlations did not demonstrate associations between frequency of AI use and social media frequency (ρ = 0.00) or between frequency of AI use and social media posting frequency (ρ = 0.00); incorporation of frequency of social media use and frequency of posting in regression models did not modify main associations of AI use (Figure 3). We also examined whether associations between AI use and mood were associated with the nature of AI use. Incorporating individual terms for school, work, and personal use, only personal use was significantly associated with PHQ-9 (β = 0.31 [95% CI, 0.10-0.52]), while the other 2 were not (Figure 4).

Finally, we investigated the possibility of differential associations between AI use and mood by gender or age. We first added an interaction term to regression models; no AI use by gender association was observed, but a significant AI by age group association was identified. We then stratified regression models by age stratum and fit individual regression models (eFigure 4 in Supplement 1). AI use was significantly associated with greater depressive symptoms among individuals aged 25 to 44 years (β = 1.22 [95% CI, 0.70-1.74]) and 45 to 64 years (β = 1.38 [95% CI, 0.72-2.05]), but not the other groups. Similarly, daily AI use was significantly associated with odds of moderate or greater depressive symptoms overall (OR, 1.29 [95% CI, 1.15-1.46]), and in individuals aged 25 to 44 years (OR, 1.32 [95% CI, 1.13-1.54]) or 45 to 64 years (OR, 1.54 [95% CI, 1.24-1.92]) (eFigure 5 in Supplement 1).

## Discussion

In this survey study including 20 847 adults from all 50 states and the District of Columbia who completed an internet survey, we found that daily AI use was common and significantly associated with depressive and other negative affective symptoms after adjusting for sociodemographic features. While the magnitudes of associations were generally modest, among individuals aged 45 to 64, the odds of reporting at least moderate depression were 50% greater for daily AI users. Given the rapidity of AI dissemination and the scale of use, these results in aggregate suggest the need to better understand potential causation and heterogeneity of outcomes.

There is little prior literature for comparison. Anecdotes regarding adverse outcomes of nonclinical chatbot use^30^ have led to efforts to ban or regulate AI use in this context.^31^ A report of chatbot interactions indicated risks associated with prolonged daily use, including diminished social interaction,^5^ similar to a pattern observed in a study of college students.^6^ Notably, briefer interactions have been associated with improvement in well-being, and a randomized trial demonstrated benefit from use of a chatbot applying a language model specifically trained to discuss mental health symptoms.^7^ Still, taken together, this literature in concert with our study suggest that more frequent use—particularly outside of work or school tasks—may be associated with poorer mental health outcomes. A further distinction in our results, other than sample size and representativeness, is the finding that the association may be heterogeneous across age groups.

This emerging picture is consistent with previous observations regarding social media use. Most of the social media literature, as with this study, is cross-sectional and correlative. Longitudinal investigations of social media use do suggest that use or abstinence may precede changes in mental health^11,12,13^. In 2 randomized clinical trials, effects of abstaining from social media use were positive but extremely modest^14,15^ and possibly specific to population subgroups or platforms. A key next step may be consideration of such approaches to the study of AI as well.

### Limitations

This study has multiple limitations. As a nonprobability internet survey in which participants from panels opt in to participation, we cannot estimate response rates and concomitant biases. The study does incorporate state-level quotas and reweighting to yield more representative samples, and prior efforts indicate consistency between our results and probability-sampled polls and administrative data.^30,32^ While differential internet access across groups is often noted as a concern for this survey approach, Pew Research data support widespread access across age, income, and education groups.^33^ A further limitation is the cross-sectional nature of these data, which does not allow us to test causation. Although our results are consistent with personal AI use causing greater depressive symptoms, they are equally consistent with greater depressive symptoms precipitating greater AI use, or with neither of these. Moreover, we cannot exclude other unmeasured confounding effects, for example from preexisting psychiatric diagnoses. Still, the ability to capture other online behavior, particularly social media use, does allow us to begin to determine the specificity of the outcomes we observed, suggesting they do not simply reflect online behavior.

## Conclusions

This survey study found that generative AI use was associated with modestly but statistically significantly greater depressive and other negative affective symptoms, warranting efforts to understand the potential for a causal relationship. Differential associations in age strata also suggest the importance of considering mechanisms underlying these subgroup associations, if some individuals may be more apt to experience depressive symptoms associated with AI use. At minimum, randomized trials examining the potential benefits of AI use should also incorporate measures of mood and anxiety along with typical assessments of productivity.

## Notes

Supplement 1. **eFigure 1.** Ordinal logistic regression model of AI use frequency **eFigure 2.** Multivariable linear regression model of anxiety symptoms **eFigure 3.** Multivariable linear regression model of irritability symptoms **eFigure 4.** Multiple linear regressions models of depression symptoms showing coefficient for daily AI use, stratified by age group

Supplement 2.
Data Sharing Statement
