# Error in Byline

**DOI:** 10.1001/jamanetworkopen.2026.2242

**Published:** 2026-02-11

In the Original Investigation titled “Generative AI Use and Depressive Symptoms Among US Adults,”^1^ published January 21, 2026, there was a typographical error in the name of coauthor Ata A. Uslu, MSc. This article has been corrected.^1^
