# Supplementary material for: Generative AI Use and Depressive Symptoms Among US Adults
Source: JAMA Netw Open. 2026 Jan 21;9(1):e2554820. doi: 10.1001/jamanetworkopen.2025.54820 (PMC12824790; doi:10.1001/jamanetworkopen.2025.54820)
Supplement: Supplement 1. — eFigure 1. Ordinal logistic regression model of AI use frequency eFigure 2. Multivariable linear regression model of anxiety symptoms eFigure 3. Multivariable linear regression model of irritability symptoms eFigure 4. Multiple linear regressions models of depression symptoms showing coefficient for daily AI use, stratified by age group [file jamanetwopen-e2554820-s001.pdf]

Supplemental Online Content

Perlis RH, Gunning FM, Uslu AA, et al. Generative AI use and depressive symptoms among US adults . *JAMA Netw Open*. 2026;9(1):e2554820. doi:10.1001/jamanetworkopen.2025.54820

eFigure 1. Ordinal logistic regression model of AI use frequency

eFigure 2. Multivariable linear regression model of anxiety symptoms

eFigure 3. Multivariable linear regression model of irritability symptoms

eFigure 4. Multiple linear regressions models of depression symptoms showing coefficient for daily AI use, stratified by age group

This supplemental material has been provided by the authors to give readers additional information about their work.

© 2026 Perlis RH et al. *JAMA Network Open*.

Supplemental Figure 1. Ordinal logistic regression model of AI use frequency

Variable		N	Estimate		p
**Gender**	Female	10324		Reference	
	Male	10386		1.38 (1.31, 1.45)	<0.001
	Nonbinary	134		1.42 (1.03, 1.96)	0.032
**Age category**	18 to 24	1855		Reference	
	25 to 44	8156		0.92 (0.84, 1.02)	0.106
	45 to 64	6650		0.69 (0.62, 0.76)	<0.001
	65 and over	4183		0.38 (0.34, 0.43)	<0.001
**Education**	Some High School or Less	755		Reference	
	High School Graduate	5618		1.33 (1.12, 1.57)	<0.001
	Some College	5104		1.84 (1.56, 2.18)	<0.001
	College Degree	6944		2.31 (1.95, 2.73)	<0.001
	Graduate Degree	2423		3.55 (2.96, 4.25)	<0.001
**Income**	Under 25K	4776		Reference	
	25k to under 50k	5376		1.25 (1.15, 1.35)	<0.001
	50K to under 100K	6539		1.41 (1.31, 1.53)	<0.001
	100K and over	4153		1.85 (1.69, 2.02)	<0.001
**Race**	Asian	980		Reference	
	Black	3865		1.00 (0.87, 1.13)	0.945
	Other	1360		0.76 (0.65, 0.90)	0.001
	White	14639		0.78 (0.70, 0.88)	<0.001
**Ethnicity**	Hispanic	2977		Reference	
	Non–Hispanic	17867		0.84 (0.77, 0.91)	<0.001
**Urbanicity**	Rural	3594		Reference	
	Suburban	11173		1.11 (1.03, 1.20)	0.007
	Urban	6077		1.30 (1.20, 1.42)	<0.001

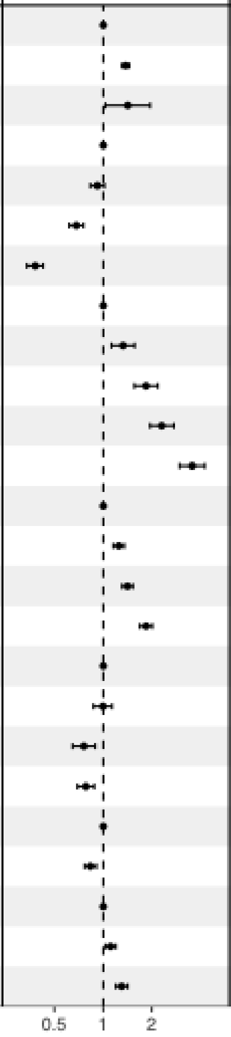

© 2026 Perlis RH et al. *JAMA Network Open*.

Supplemental Figure 2. Multivariable linear regression model of anxiety symptoms (GAD-2)

Variable		N		p
**Frequency of AI use**	Never	11336	Reference	
	Once or twice	2890	0.08 (−0.01, 0.16)	0.07
	About once a month	1227	0.08 (−0.04, 0.21)	0.19
	About once a week	1407	0.13 (0.02, 0.24)	0.02
	Multiple times a week	1832	0.12 (0.02, 0.23)	0.02
	Every day	1053	0.25 (0.11, 0.39)	<0.001
	Multiple times a day	1099	0.30 (0.16, 0.44)	<0.001
**Gender**	Female	10324	Reference	
	Male	10386	−0.33 (−0.39, −0.27)	<0.001
	Nonbinary	134	1.12 (0.71, 1.52)	<0.001
**Age category**	18 to 24	1855	Reference	
	25 to 44	8156	−0.24 (−0.36, −0.13)	<0.001
	45 to 64	6650	−0.78 (−0.90, −0.66)	<0.001
	65 and over	4183	−1.48 (−1.60, −1.36)	<0.001
**Education**	Some High School or Less	755	Reference	
	High School Graduate	5618	−0.18 (−0.35, −0.01)	0.04
	Some College	5104	−0.16 (−0.33, 0.01)	0.06
	College Degree	6944	−0.34 (−0.51, −0.17)	<0.001
	Graduate Degree	2423	−0.32 (−0.50, −0.13)	<0.001
**Income**	Under 25K	4776	Reference	
	25k to under 50k	5376	−0.18 (−0.27, −0.10)	<0.001
	50K to under 100K	6539	−0.40 (−0.49, −0.32)	<0.001
	100K and over	4153	−0.59 (−0.68, −0.49)	<0.001
**Race**	Asian	980	Reference	
	Black	3865	0.02 (−0.11, 0.16)	0.71
	Other	1360	0.31 (0.13, 0.48)	<0.001
	White	14639	0.45 (0.32, 0.57)	<0.001
**Ethnicity**	Hispanic	2977	Reference	
	Non−Hispanic	17867	−0.00 (−0.10, 0.09)	0.93
**Urbanicity**	Rural	3594	Reference	
	Suburban	11173	0.09 (0.01, 0.17)	0.02
	Urban	6077	0.09 (0.00, 0.18)	0.05

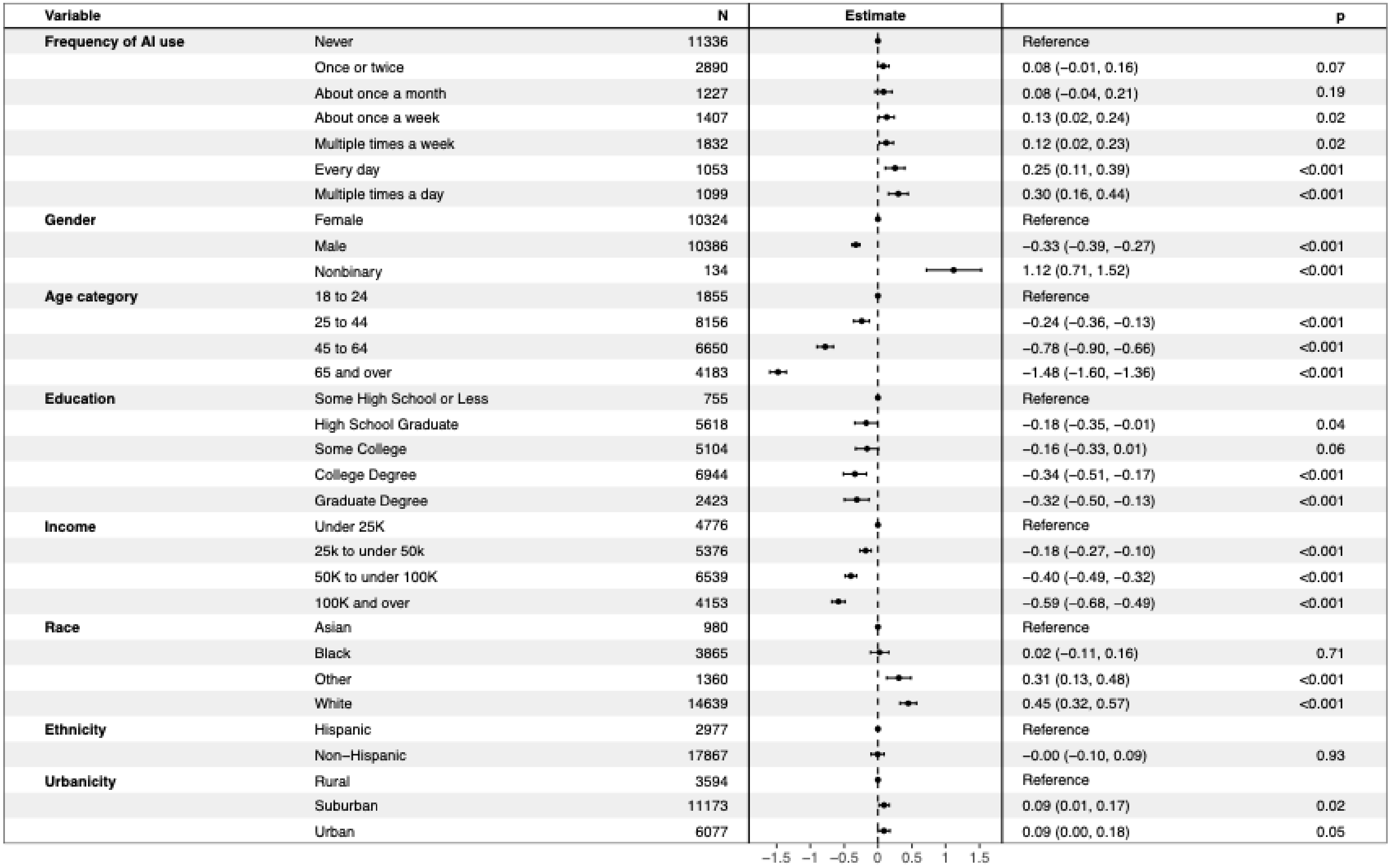

© 2026 Perlis RH et al. *JAMA Network Open*.

Supplemental Figure 3. Multivariable linear regression model of irritability symptoms (BITe-5)

Variable		N	Estimate	p
**Frequency of AI use**	Never	11336	Reference	
	Once or twice	2890	0.19 (−0.06, 0.44)	0.145
	About once a month	1227	0.50 (0.10, 0.89)	0.013
	About once a week	1407	0.17 (−0.17, 0.51)	0.319
	Multiple times a week	1832	0.03 (−0.30, 0.36)	0.857
	Every day	1053	0.75 (0.29, 1.22)	0.001
	Multiple times a day	1099	0.71 (0.23, 1.19)	0.004
**Gender**	Female	10324	Reference	
	Male	10386	−0.67 (−0.85, −0.49)	<0.001
	Nonbinary	134	1.87 (0.68, 3.06)	0.002
**Age category**	18 to 24	1855	Reference	
	25 to 44	8156	−0.54 (−0.91, −0.18)	0.004
	45 to 64	6650	−2.22 (−2.59, −1.84)	<0.001
	65 and over	4183	−5.23 (−5.60, −4.85)	<0.001
**Education**	Some High School or Less	755	Reference	
	High School Graduate	5618	−0.86 (−1.39, −0.33)	0.001
	Some College	5104	−0.95 (−1.48, −0.42)	<0.001
	College Degree	6944	−1.64 (−2.17, −1.11)	<0.001
	Graduate Degree	2423	−1.40 (−1.97, −0.82)	<0.001
**Income**	Under 25K	4776	Reference	
	25k to under 50k	5376	−0.36 (−0.63, −0.09)	0.008
	50K to under 100K	6539	−0.79 (−1.05, −0.53)	<0.001
	100K and over	4153	−1.20 (−1.50, −0.89)	<0.001
**Race**	Asian	980	Reference	
	Black	3865	0.41 (−0.05, 0.86)	0.078
	Other	1360	1.01 (0.43, 1.60)	<0.001
	White	14639	1.23 (0.82, 1.63)	<0.001
**Ethnicity**	Hispanic	2977	Reference	
	Non−Hispanic	17867	−0.08 (−0.39, 0.22)	0.586
**Urbanicity**	Rural	3594	Reference	
	Suburban	11173	−0.01 (−0.25, 0.22)	0.911
	Urban	6077	−0.15 (−0.42, 0.13)	0.289

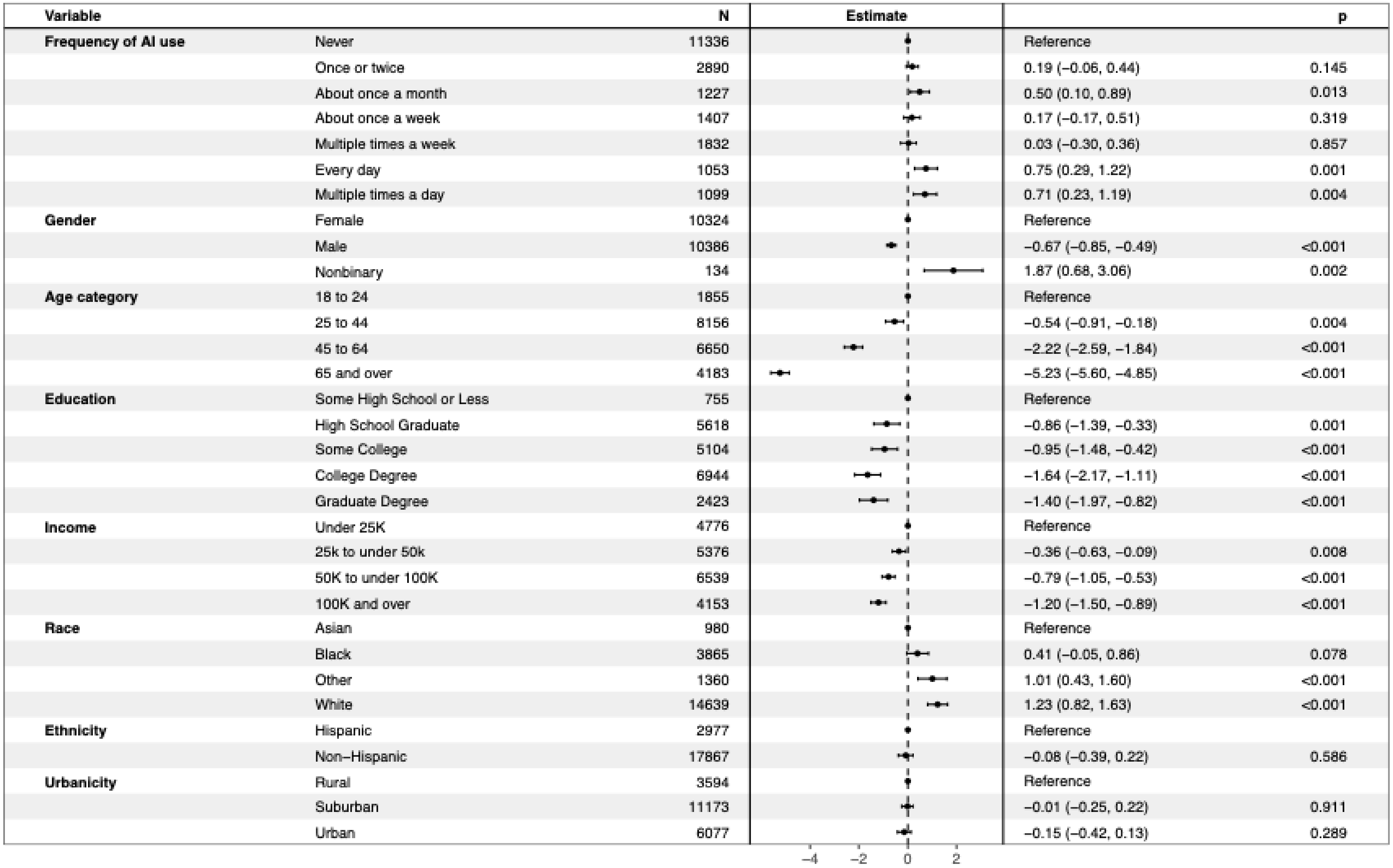

© 2026 Perlis RH et al. *JAMA Network Open*.

Supplemental Figure 4. Multiple linear regression models of depressive symptoms (PHQ-9) showing coefficient for daily AI use, stratified by age group

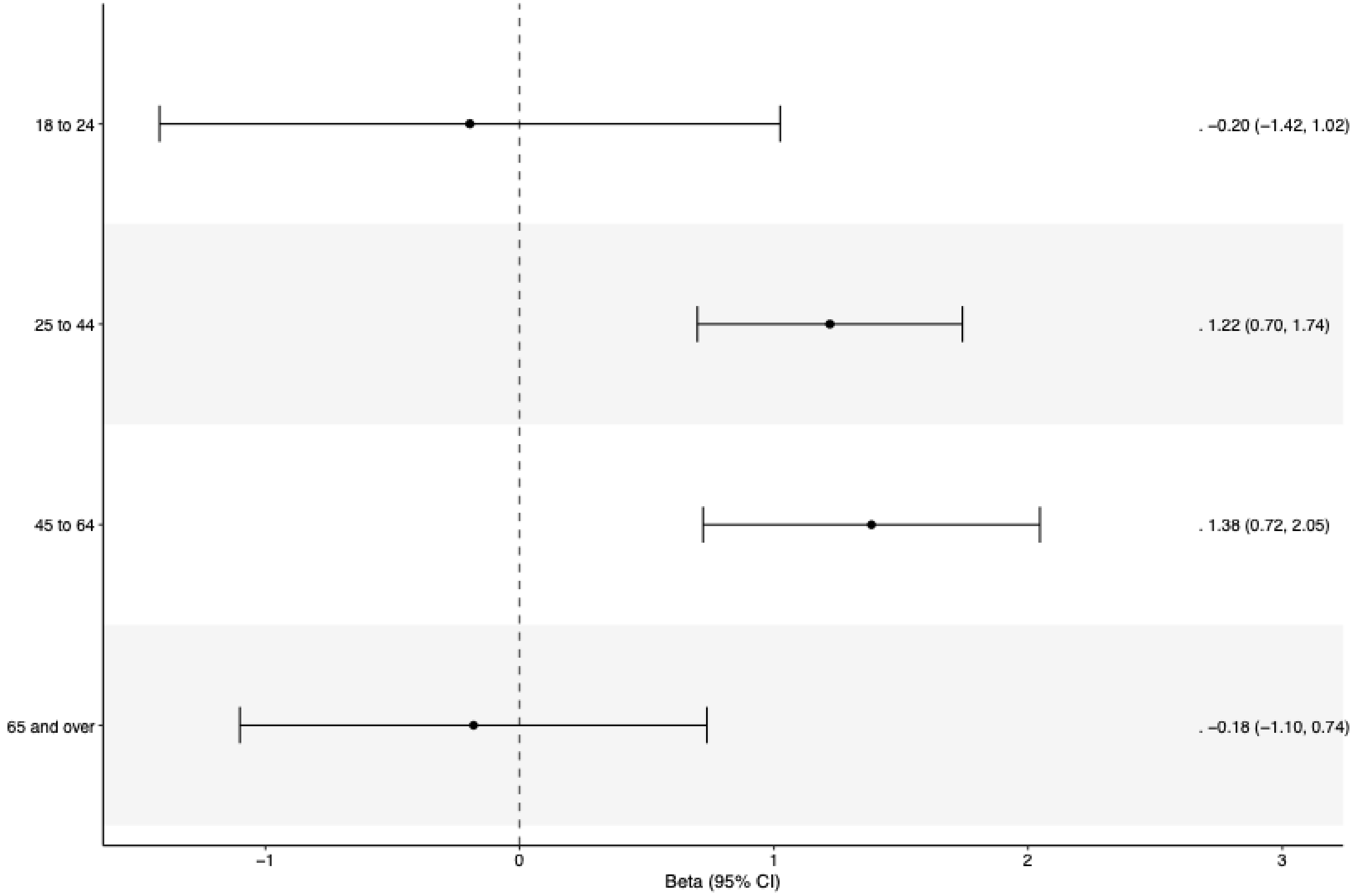

© 2026 Perlis RH et al. *JAMA Network Open*.

Supplemental Figure 5. Coefficients from individual multiple logistic regression model of association between at least daily AI use and moderate or greater depressive symptoms, adjusted for sociodemographic features and stratified by age group

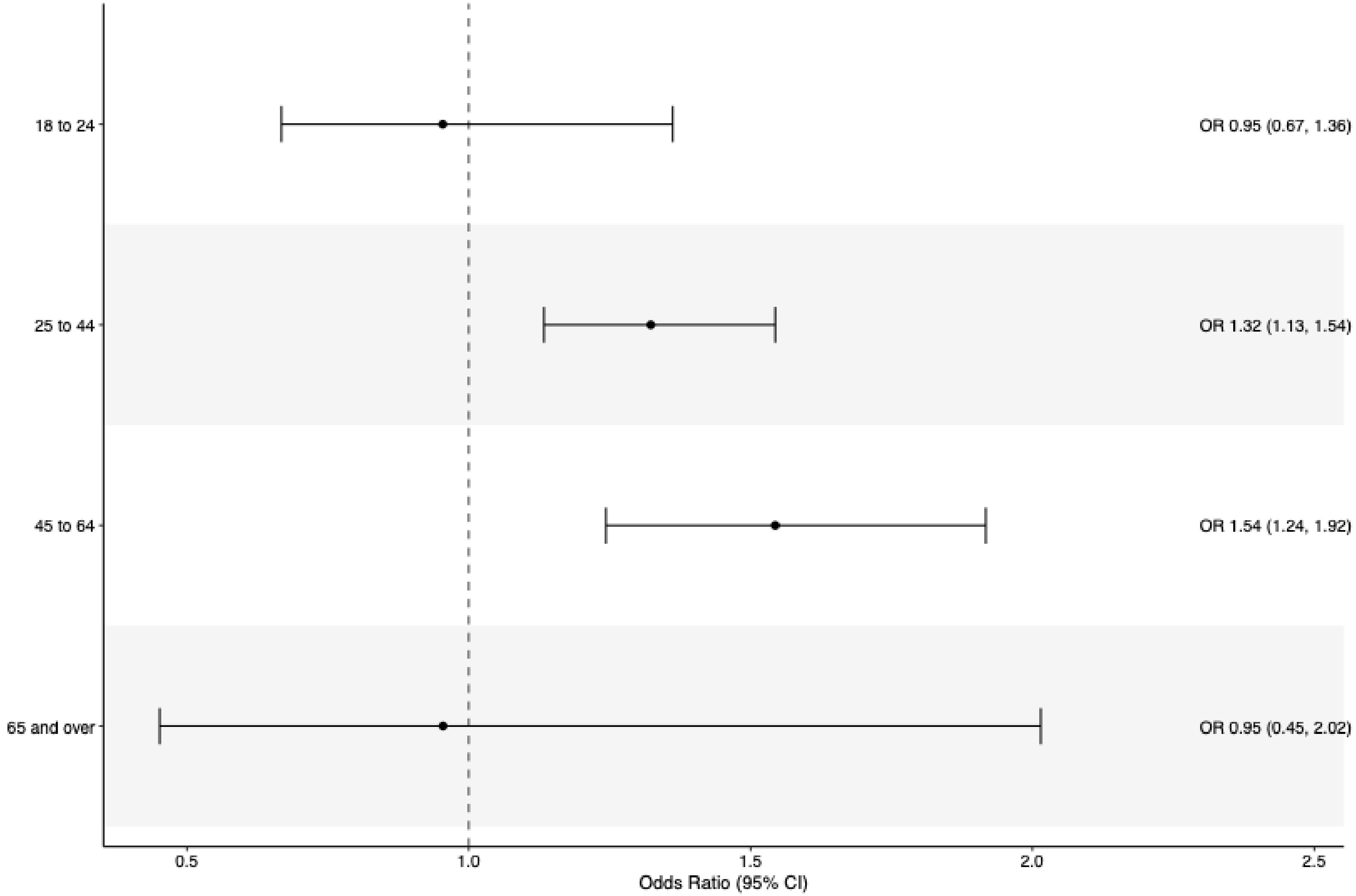

© 2026 Perlis RH et al. *JAMA Network Open*.
