# Supplementary material for: Generative AI Use and Depressive Symptoms Among US Adults
Source: JAMA Netw Open. 2026 Jan 21;9(1):e2554820. doi: 10.1001/jamanetworkopen.2025.54820 (PMC12824790; doi:10.1001/jamanetworkopen.2025.54820)
Supplement: Supplement 2. — Data Sharing Statement [file jamanetwopen-e2554820-s002.pdf]

Data Sharing Statement

Perlis. Generative AI Use and Depressive Symptoms Among US Adults. *JAMA Netw Open*. Published January 21, 2026. doi:10.1001/jamanetworkopen.2025.54820

Data
Data available: Yes
Data types: Deidentified participant data
How to access data: via the study web site, for noncommercial use by researchers upon request
When available: With publication

Supporting Documents
Document types: None

Additional Information
Who can access the data: via the study web site, for noncommercial use by researchers upon request
Types of analyses: via the study web site, for noncommercial use by researchers upon request
Mechanisms of data availability: via the study web site, for noncommercial use by researchers upon request
Any additional restrictions: via the study web site, for noncommercial use by researchers upon request
